# Visualisation of experimentally determined and predicted protein N-glycosylation and predicted glycosylphosphatidylinositol anchor addition in *Trypanosoma brucei*.

**DOI:** 10.12688/wellcomeopenres.17640.1

**Published:** 2022-01-31

**Authors:** Michele Tinti, Michael A. J. Ferguson

**Affiliations:** 1 Wellcome Centre for Anti-Infectives Research (WCAIR), School of Life Sciences, University of Dundee, Dundee, Scotland, DD1 5HN, UK

**Keywords:** Trypanosoma brucei, proteomics, glycobiology, N-glycosylation, glycosylphosphatidylinositol, oligosaccharyltransferase, OST, prediction

## Abstract

**Background:** *Trypanosoma brucei *is a protozoan parasite and the etiological agent of human and animal African trypanosomiasis. The organism cycles between its mammalian host and tsetse vector. The host-dwelling bloodstream form of the parasite is covered with a monolayer of variant surface glycoprotein (VSG) that enables it to escape both the innate and adaptive immune systems. Within this coat reside lower-abundance surface glycoproteins that function as receptors and/or nutrient transporters. The glycosylation of the *Trypanosoma brucei *surface proteome is essential to evade the immune response and is mediated by three oligosaccharyltransferase genes; two of which, TbSTT3A and TbSTT3B, are expressed in the bloodstream form of the parasite.

**Methods: **We processed a recent dataset of our laboratory to visualise putative glycosylation sites of the Trypanosoma brucei proteome. We provided a visualisation for the predictions of glycosylation carried by TbSTT3A and TbSTT3B, and we augmented the visualisation with predictions for Glycosylphosphatidylinositol anchoring sites, domains and topology of the Trypanosoma brucei proteome.

**Conclusions: **We created a web service to explore the glycosylation sites of the Trypanosoma brucei oligosaccharyltransferases substrates, using data described in a recent publication of our laboratory. We also made a machine learning algorithm available as a web service, described in our recent publication, to distinguish between TbSTT3A and TbSTT3B substrates.

## Introduction

The protozoan parasite *Trypanosoma brucei* is transmitted to humans by the tsetse fly (Glossina species), which is found only in sub-Saharan Africa ^1^. The parasite replicates as procyclic form (PCF) in the tsetse fly midgut and some differentiate during migration to the salivary glands to replicating epimastigote forms. The latter differentiate into non-dividing metacyclic trypomastigotes that establish the mammalian host infection during a tsetse vector bloodmeal. Once in the host, the parasites differentiate into replicating, slender trypomastigotes and some of these differentiate into non-dividing stumpy forms that are adapted for survival and differentiation into procyclic forms once ingested by the vector ^1^. Left untreated in the human host, the parasites invade the central nervous system causing neurological symptoms, coma and death ^1^. The majority of experimental data on *T. brucei* have been obtained from either cultured versions of the bloodstream form (BSF), or BSF cells from rodent infections, and/or from the cultured procyclic form (PCF) of the parasite.

Like all eukaryotes, *T. brucei* modifies most proteins that enter its secretory pathway through glycosylation. Since cell surface glycoproteins are at the interface between the cell and its environment, they often play central roles in eukaryotic cell biology; *T. brucei* is no exception. The BSF relies on a surface coat made of glycosylphosphatidylinositol (GPI) anchored and *N-*glycosylated variant surface glycoprotein (VSG) to evade the host innate immune system and the acquired immune system through antigenic variation ^2^. The BSF also expresses other lower abundance glycoproteins including but not restricted to: a novel VSG-like transferrin receptor (TfR) ^2– 4^, a lysosomal/endosomal protein called p67 ^5^, invariant surface (ISG) and endoplasmic reticulum (IGP) glycoproteins ^6, 7^, a Golgi/lysosomal glycoprotein tGLP-1 ^8^, a membrane-bound histidine acid phosphatase TbMBAP1 ^9^, flagellar adhesion zone glycoproteins Fla1–3 ^10, 11^, a flagellar pocket/endosomal system haptoglobin-hemoglobin receptor (HpHbr) ^12^ and serum resistance antigen (SRA) ^13^, a complement factor H receptor (FHR) ^14^ and a metacyclic trypomastigote-specific ISG ^15^. Some of these are metacyclic and/or BSF specific glycoproteins (eg. VSG, TfR, ISG, TbMAP1, HpHbr, SRA, FHR) while others are also common to PCF trypanosomes. PCF parasites also express unique glycoproteins including but not limited to: the abundant GPI-anchored procyclins, some of which are *N*-glycosylated ^16, 17^, and a high-molecular weight glycoconjugate ^18, 19^.

The GPI anchor structures of some BSF VSGs ^20– 23^ and the TfR ^24^ have been solved, as have those of PCF procyclins ^16^. All contain the conserved GPI core but the BSF GPIs contain sn-1,2-dimyristoylglycerol lipid and sidechains of up to 1 βGal and up to 5 αGal residues whereas the PCF procyclin GPIs are inositol-acylated and contain sn-1-acylglycerol lipid and sidechains of branched, *N*-acetyllactosamine and lacto- *N*-biose repeats capped with α2–3 sialic acid ^16, 25, 26^. Expression of a BSF VSG gene in PCF cells resulted in PCF-type GPI anchor inositol-acylation and sidechain structure ^27^. We therefore conclude that *T. brucei* GPI anchors can be categorized as BSF- or PCF-type according th the lifecycle stage they are expressed in.

Several of the *N-*glycan structures expressed by BSF *T. brucei* have been solved and these include conventional oligomannose and biantennary complex structures as well as paucimannose and extremely unusual ‘giant’ poly- *N-*acetyl-lactosamine (poly-LacNAc) containing complex structures ^28– 32^. In contrast, only oligomannose *N*-glycans have been structurally described in wild type PCF trypanosomes ^16, 33^. Eukaryotic oligosaccharyltransferase (OST) enzymes responsible for N-glycosylation operate on asparagine residues in N-glycosylation sequon motifs of asparagine, any amino acid except proline, serine or threonine (N.^P[S/T]). We showed that two OST enzymes in BSF *T. brucei*, named TbSTT3A and TbSTT3B, have different acceptor and donor substrate specificities ^33^. Thus, TbSTT3A first transfers Man5GlcNAc2 from Man5GlcNAc2-PP-dolichol to any sequons in acidic peptide environments and TbSTT3B transfers Man9GlcNAc2 from Man9GlcNAc2-PP-dolichol to all remaining sequons. The sites modified by TbSTT3A with bi-antennary Man5GlcNAc2 can be further processed to paucimannose structures and a wide array of complex N-glycan structures, while the sites modified by TbSTT3B with tri-antennary Man9GlcNAc2 can be maximally processed to tri-antennary Man5GlcNAc2; i.e., these sites are exclusively occupied by oligomannose N-glycans. Using this information, we were able to create a predictor to distinguish between N-glycosylation sequons preferentially modified by TbSTT3A, leading to paucimannose and/or complex N-glycans, or TbSTT3B, leading it oligomannose N-glycans ^33^. Experimental proteomics data used to train the predictor exploited the sensitivity and resistance, respectively, of oligomannose and paucimannose/complex N-glycans. Removal of oligomannsoe glycans by endoglycosidase H leaves behind a single N-acetylglucosamine residue and thus marks relevant tryptic peptides with a 203 D mass-tag. The endoglycosidase H resistant paucimannose/complex N-glycans were subsequently removed with peptide N-glycosidase F in the presence of H _2_^18^O, leaving behind [ ^18^O]aspartate in place of asparagine and thus marking relevant tryptic peptides with a 3 D mass-tag.

To facilitate the visualisation and analysis of putative *T. brucei* glycoproteins based on their predicted amino acid sequences, we have combined the prediction of N-terminal signal peptides (that are generally required for protein entry into the secretory pathway), C-terminal GPI addition signal peptides, N-glycosylation sequon (classified as experimentally determined and/or predicted TbSTT3A or TbSTT3B substrates) transmembrane and other protein domains. We have created a free to use web service incorporating all these features that we believe will be useful to the trypanosome research community.

## Methods

We used the mass spectrometry data described in 33 and deposited at the PRIDE database ^34^ with accession numbers: PXD007267 and PXD007268 to extract the BSF glycoprotein sequons preferentially modified by TbSTT3A (and therefore expressing complex and/or paucimannose N-glycans) or TbSTT3B (and therefore expressing oligomannose N-glycans). We also computed the ratio of the complex/paucimannose modifications as TbSTT3A modified sites / (TbSTT3A modified sites + TbSTT3A modified sites). Similarly, we computed the ratio of the oligomannose modifications as TbSTT3B modified sites / (TbSTT3B modified sites + TbSTT3A modified sites). We also collected transmembrane topology and signal peptide predictions using the Phobius website https://phobius.sbc.su.se/index.html ^35^ and GPI anchor site predictions using the big-PI Predictor available at https://mendel.imp.ac.at/gpi/gpi_server.html ^36^. The machine-learning algorithm to distinguish the sites preferentially modified by TbSTT3B or TbSTT3A in BSF *T. brucei* is the same described in 33. We further collected protein domain predictions using the CDART server ^37^. The protein ids, sequences and descriptions were retrieved from TriTrypDB version 28 ^38^. TriTrypDB stores also user-based comments regarding the gene of interest and gene ontology (GO) annotation terms that were also retrieved and incorporated in the web application.

### Implementation

We implemented a web server using the tornado python package version 4.3 ( https://www.tornadoweb.org/en/stable/). The user interface was developed in javascript using bootstrap version 3.3.7, jquery version 3.1.1 and datatables version 1.10.11. The feature visualisation panel uses the neXtProt feature viewer package version 0.1.44 ^39^. The website is hosted at http://134.36.66.166:8070/home.

### Operation

We recommend hosting the application on a web server with 1MB of RAM and 50GB of disk space. The application runs using the Tornado HTTPServer ( https://www.tornadoweb.org/en/stable/guide/running.html). The application code can be cloned from the git repository or downloaded from Zenodo ^40^. After creating and activating a conda environment with the packages listed in requirments.txt ^40^, move to the application folder and start the Tornado HTTPServer with “python glyc_web_server.py”

## Use cases

**Figure 1.**
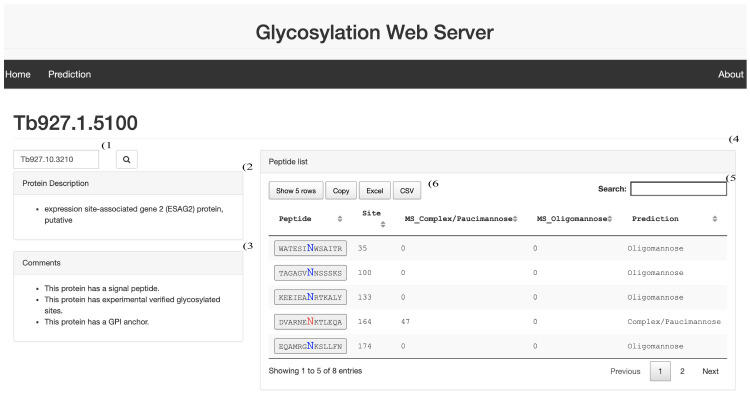
Web Application Layout. Screen shot of the upper half of the web application user interface. 1) Input text to query the web server with a protein identification number. 2) Text area reporting the protein description. 3) Text area reporting the presence of three protein features: Signal peptide, Glycosylation sites and GPI anchor. 4) Tab reporting the N-glycan peptide sequences identified in the protein sequence. 5) Search field for the peptide sequences. 6) Download buttons for the table listing the peptide sequences.

**Figure 2.**
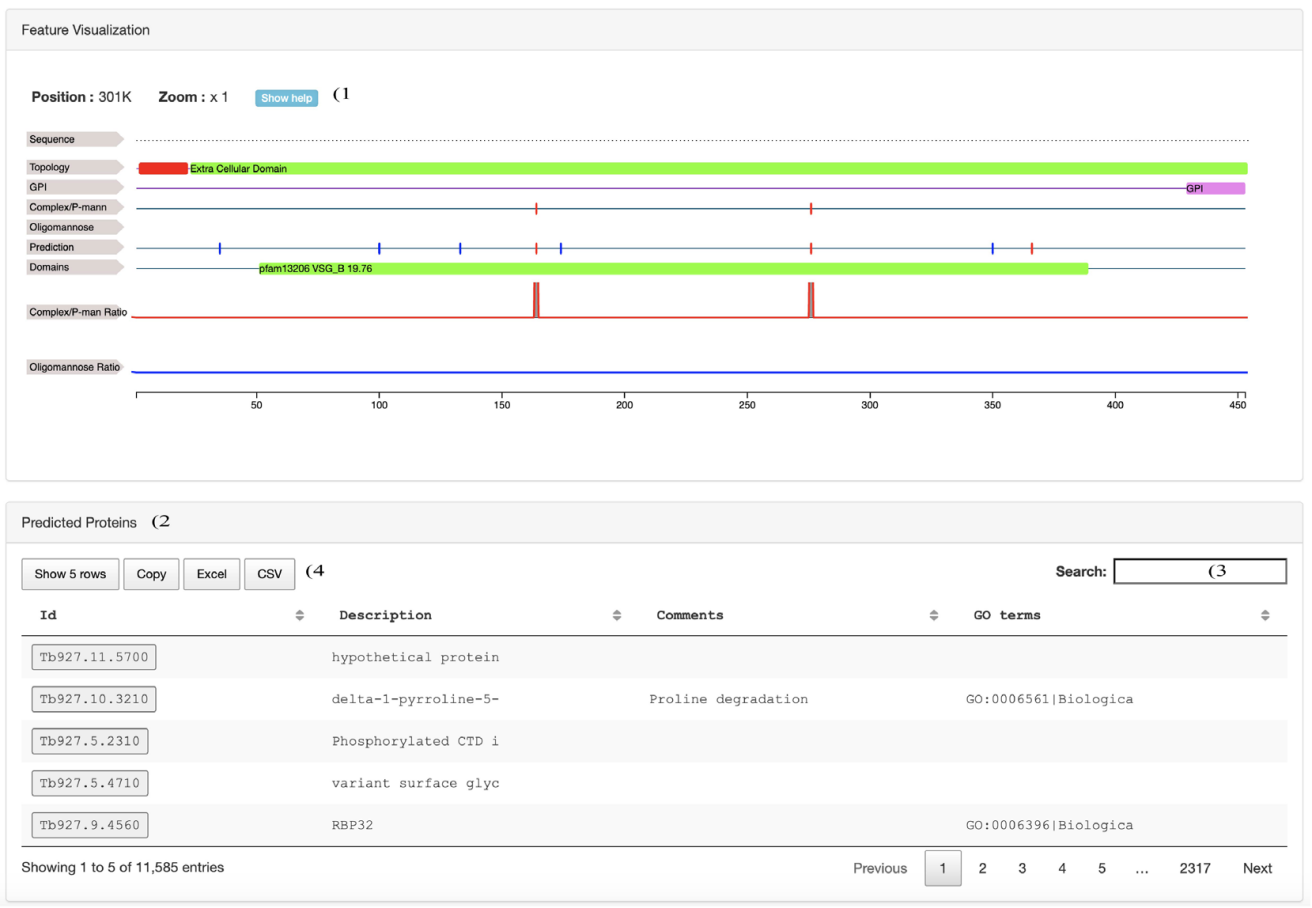
Web Application Layout. Screen shot of the bottom half of the web application user interface. 1) Feature visualization panel for the selected protein 2) Protein table listing the protein identification number available in the web application. 3) Search field for the protein table 4) Download buttons for the protein table.

**Figure 3.**
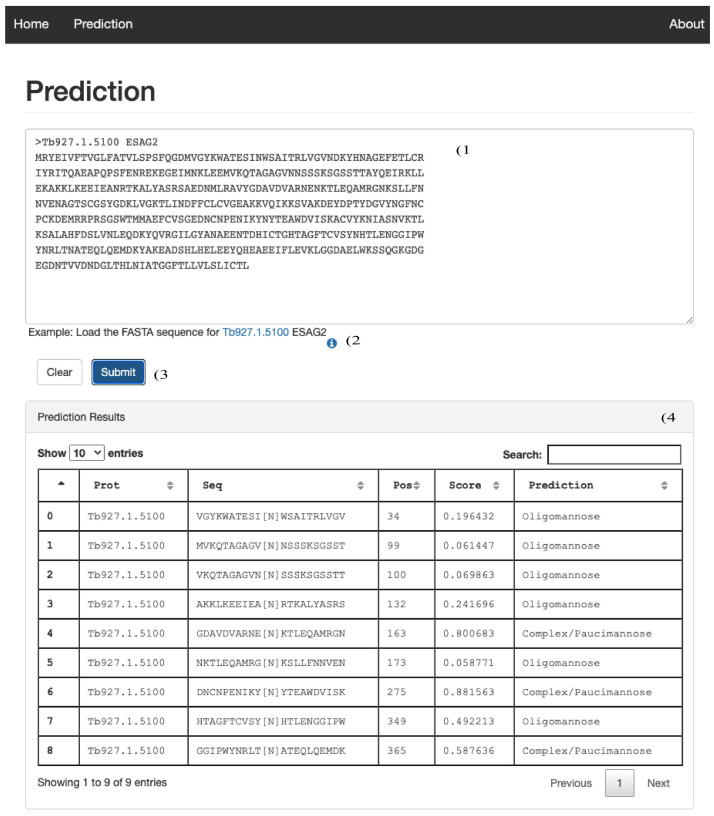
Protein Prediction page. Screen shot of the user interface to submit a protein sequence for predictions. 1) Input text area to copy\paste a protein sequence in FASTA format. 2) Submit button to start the prediction. 3) Text area to be populated with the prediction output.

The user is presented with a responsive web application with two main components: a protein feature browser ( Figure 1 and Figure 2) and a type of glycans prediction ( Figure 3).

### Protein feature browser

The protein feature browser can be queried with a protein identification number ( Figure 1.1). After clicking the search button, the protein description and comments tab are updated ( Figure 1.2 and Figure 1.3). The comment tab reports on the presence of: 1) a signal peptide, 2) the presence of occupied N-glycosylation sequons, as determined by mass spectrometry, and 3) the presence of a predicted GPI anchoring site. The peptide list tab ( Figure 1.4) reports all the N.^P[S/T] sequons identified in the protein. It reports the peptide sequence (peptide) extracted from +/- 6 amino acid surrounding the central asparagine. The central asparagine is colour coded blue if predicted to be be modified by TbSTT3B, and therefore carry olgomannose N-glycans, or red if predicted to be be modified by TbSTT3A in BSF cells, and therefore carry paucimannose or complex N-glycans, as reported in the Prediction column. The table further reports the N-glycan occupied site position in the protein sequence (Site), the number of peptides detected by mass spectrometry indicating they were originally occupied by endoglycosidse H-resistant complex/paucimannose glycans (MS_complex / paucimannose), the number of peptides detected by mass spectrometry indicating they were originally occupied by endoglycosidse H-sensitive oligomannose glycans (MS_oligomannose). The table can be searched by peptide sequence or prediction type with the Search input field ( Figure 1.5). The table can also be downloaded locally with the interaction buttons ( Figure 1.6). 

The protein identification number search button ( Figure 1.2) also updates the visual protein sequence representation in the central part of the web page ( Figure 2.1) reporting: 1) the protein sequence (Sequence), 2) the protein region predicted to be cleaved off after the addition of the GPI anchoring site (GPI), 3) The localisation of complex/paucimannose glycans identified by mass spectrometry, 4) The localisation of oligomannose glycans identified by mass spectrometry, 5) the CDART protein domain predictions, 6) the proportion of complex/paucimannose modifications and 7) the proportion of of the oligomannose modifications.

The full dataset hosted in the web application can be queried with the table at the bottom of the web application ( Figure 2.2). The table can be searched using the search field ( Figure 2.3) with the protein identification number (Id), gene description (Description), user-defined comments (Comments) and GO term annotations (GO term). The table can also be downloaded locally with the interaction buttons ( Figure 2.4).

### N-Glycan type prediction

The prediction link opens another user interface where it is possible to retrieve the prediction of a machine learning model trained to discriminate between sites preferentially modified by TbSTT3A (complex/paucimannose) or TbSTT3B (oligomannose) in BSF trypanosomes. The user can input a protein sequence in Fasta format ( Figure 3.1), or an example sequence in Fasta format can be uploaded in the text input area by clicking on the Tb927.1.5100 protein id ( Figure 3.2). After clicking on the Submit button ( Figure 3.3) a results table is produced ( Figure 3.4) reporting 1) the protein identification number (Prot), 2) The putative N.^P[S/T] sites in the protein as a peptide sequence (Seq) centred at the modified asparagine +/- 10 amino acids, 3) the predictor score (Score) and 4) the type of prediction (Prediction); Oligomannose glycans for TbSTT3B modified asparagine or Complex/Paucimannose glycans for TbSTT3A modified sites. The predictor was developed as a binary classifier for TbSTT3A modified sites using TbSTT3B modified as a negative set ^33^. For this reason, a score close to 1 is indicative of a site preferentially modified by TbSTT3A. A score close to 0 is indicative of a site preferentially modified by TbSTT3B. A cut-off of 0.5 is used to determine if TbSTT3A or TbSTT3B is predicted to preferentially modify the asparagine.

## Conclusions

We developed a web application to explore the glycosylation modifications mediated by TbSTT3A and TbSTT3B in the BSF proteome of *T. brucei.* It is important to re-emphasise that in wild type PCF *T. brucei*, only oligomannose N-glycans have been described and that this is largely controlled by suppression of TbSTT3A expression in that lifecycle stage. Thus, every occupied N-glycosylation sequon in wild type PCF cells is predicted to be of the oligomannose type.

It is also worth noting that the predictions that we present classify every asparagine in embedded in a N.^P[S/T] motif, even if it is biologically unlikely. For example, the predicted asparagine might reside in a protein that lacks an N-terminal signal peptide, or reside in a transmembrane region, in a signal peptide region or in the region excised after GPI modification of a protein. For this reason, we augmented our predictions with several visualisations of protein sequence properties (signal peptide, topology and GPI) predicted from other web services ^35– 37^). This should allow the interested user to evaluate both the type of glycan modifications and its biological relevance for the predicted sites.

## Software availability

Source code available from: https://github.com/mtinti/glycosylation-server.

Archived source code at time of publication: https://doi.org/10.5281/zenodo.5878703 ^40^.

License: MIT.

Zenodo: mtinti/glycosylation-server: v0.1.

This project contains the following data:

- asap
  Python code to extract features from peptide sequence
- data
  Files to store pre-computed protein features
- models
  The model used for the glycosylation prediction
- scripts
  python code to parse protein features
- static
  javascript codes for the web server
- templates
  HTML code for the web server
- glyc_web_server.py
  Python code to start the web server
- predict_seq.py
  Python helper functions for the prediction page of the web server
- protein.py
  Python helper functions for the web server
- requirements.txt
  List of python packages to run the web server
