# Supplementary material for: Visualisation of experimentally determined and predicted protein N-glycosylation and predicted glycosylphosphatidylinositol anchor addition in Trypanosoma brucei
Source: Wellcome Open Res. 2022 Jan 31;7:33. [Version 1] doi: 10.12688/wellcomeopenres.17640.1 (PMC8886175; doi:10.12688/wellcomeopenres.17640.1)
Supplement: Peer review history

# Peer review: Reviewer response for version 1

**DOI:** 10.21956/wellcomeopenres.19514.r48799

**Published:** 2022-03-09

**Authors:** Emma M. Briggs

**Affiliations:** 1 Institute for Immunology and Infection Research, School of Biological Sciences, University of Edinburgh, Edinburgh, UK; 2 Wellcome Centre for Integrative Parasitology, University of Glasgow, Glasgow, UK

The authors present a web interface to allow users to explore the results of several prediction algorithms, including their own to predict *N-*glycosylation sites and discriminate TbSTT3A and TbSTT3B targets.

 The web interface is functional and clear to use and will be useful to allow others to explore protein features. 

 The authors describe the use of Phobius, big-PI, and CDART for other predicted protein features. The parameters used for these can be included in the methods, even if all defaults were used. Which Phobius predictor was used (normal, constrained and homology supported predictions are all options on the referenced website). 

 Discussion of an experimentally validated prediction would be useful to assess the accuracy of predicted N *-*glycosylation sites, and other features. Reference 33 does not appear to have experimentally validated N *-*glycosylation predictions either. 

 Nevertheless, the web tool presented here has successfully enabled the community to make use of these predictions. 

**Typos:**

- "TbSTT3A modified sites / (TbSTT3A modified sites + TbSTT3A modified sites)"should these all be 3A?
- "N-glycans" - N is sometimes in italics and sometimes not. 

Are the conclusions about the tool and its performance adequately supported by the findings presented in the article?

Partly

Is the rationale for developing the new software tool clearly explained?

Yes

Is the description of the software tool technically sound?

Yes

Are sufficient details of the code, methods and analysis (if applicable) provided to allow replication of the software development and its use by others?

Partly

Is sufficient information provided to allow interpretation of the expected output datasets and any results generated using the tool?

Yes

Reviewer Expertise:

Molecular parasitology, single cell transcriptomics, bioinformatics, trypansomes.

I confirm that I have read this submission and believe that I have an appropriate level of expertise to confirm that it is of an acceptable scientific standard.

# Peer review: Reviewer response for version 1

**DOI:** 10.21956/wellcomeopenres.19514.r48597

**Published:** 2022-02-28

**Authors:** Michael P. Barrett

**Affiliations:** 1 Glasgow Polyomics, College of Medical, Veterinary and Life Sciences, University of Glasgow, Glasgow, UK

The submitted manuscript provides a succinct pointer towards a website and software tool allowing prediction of potential N-glycosylation sites related to the oligosaccharide transferases TbSST3A and TbSST3B in BSF T. brucei. The site is easily accessed and very fast. The code is accessible and can be downloaded too.

 Very trivially, please correct the spelling of oligomannsoe 5 lines up in paragraph 4 of the Intro.

 The one area I think important to consider though, as with any open access software offering, is how to future proof it. One solution would be to integrate into TritrypDB and it would be worth contacting the EuPathDB team to discuss doing so. This could also help with another possible risk around changing accession numbers. Here TritrypDB version 28 has been used to bring query sequences directly through the algorithm. Linking the predictions to existing proteomics datasets that have previously been derived to seek for experimentally demonstrated N-glycosylation is a very positive addition to the software. However, this can bring problems where accession numbers change (and indeed one has to be careful of strain use and also isoform differences).  For example, given the previous work Mehlert *et al. *2012, PloS Pathogen ^1^) on TfR N-glycosylation, this would be a good exemplar with which to see how the software performs.  In haste, I pulled out the first visible ESAG6 and ESAG7 entries in tritrypDB and plugged those accession numbers in.  For ESAG6, 5 predicted sites came out, but no MS hits were detected.  For EASG7, 2 predicted sites came out, and no MS hits.  As there are multiple isoforms of ESAG6 and ESAG 7 and sequences from multiple strains are present in tritrypDB matching the published and extracted sequences adds a layer of complexity.  This particular example could be a good case study to include in the manuscript here to help users know how best to navigate the system.

 For the uninitiated, it would also be useful to have a comment in the Introduction about the kinds of proteins likely to be N-glycosylated by TbSTT3A and 3B. I checked numerous transporters and enzymes in which I have a particular interest and many have predicted sites, but none show up in the MS datasets, presumably because it is membrane-destined proteins that originate in the ER that are substrates (hence the inclusion of information on the likely presence of signal peptides is useful, as are the other parts of information included in the visualisations). However, if, for example, potential N-glycosylation via TbSTT3A and B did become a feature in tritrypDB many false positive predictions will arise on possible sites, hence some clear qualifying prose here about protein types most likely to be true substrates would be helpful.

Are the conclusions about the tool and its performance adequately supported by the findings presented in the article?

Yes

Is the rationale for developing the new software tool clearly explained?

Yes

Is the description of the software tool technically sound?

Yes

Are sufficient details of the code, methods and analysis (if applicable) provided to allow replication of the software development and its use by others?

Yes

Is sufficient information provided to allow interpretation of the expected output datasets and any results generated using the tool?

Partly

Reviewer Expertise:

Biochemical Parasitology

I confirm that I have read this submission and believe that I have an appropriate level of expertise to confirm that it is of an acceptable scientific standard.

## References

[1] : Modeling of the N-glycosylated transferrin receptor suggests how transferrin binding can occur within the surface coat of Trypanosoma brucei. *PLoS Pathog* .2012;8(4) : 10.1371/journal.ppat.1002618 e1002618 10.1371/journal.ppat.1002618 22496646PMC3320590

# Peer review: Reviewer response for version 1

**DOI:** 10.21956/wellcomeopenres.19514.r48420

**Published:** 2022-02-02

**Authors:** James D. Bangs

**Affiliations:** 1 Department of Microbiology and Immunology, Jacobs School of Medicine and Biomedical Sciences, University at Buffalo (SUNY), Buffalo, NY, USA

This short manuscript describes a newly developed website for in silico analyses of N-glycosylation in *Trypanosoma brucei.* The background for this is that the bloodstream form of this parasite adds distinct glycan structures (oligo- vs. pauci-mannose) based on two distinct oligosaccharyltransferases with different specificity for acidic or neutral/basic sequons. The underlying database is populated with experimentally determined site-specific data, and where this is absent, predictions based on the above-mentioned OST specificities. This reviewer spent several hours test driving the site with the highly glycosylated lysosomal transmembrane protein p67. It was very fun. This website will be useful for experienced glycobiologists and novices alike. There are several minor comments:

1. I believe that the passage on page 4 (column 1, first paragraph, lines 4-5) that reads: “TbSTT3A modified cites / (TbSTT3 **A** modified sites + TbSTT3A modified sites)” should read: “TbSTT3A modified cites / (TbSTT3 **B** modified sites + TbSTT3A modified sites)”.
2. Suggestion: Add sequon pI on the ‘features’ list? Not necessary but might correlate with sites that are mixed pauci/oligomannose.
3. I had a different layout on the p67 ‘features visualization’ section than the one you show in Fig 2. There are several extra lines, which I found useful, that are not in the example shown. To compare query Tb927.5.1810. May want to replace the example in the figure.
4. Zoom: I don’t use double click on my mouse and I found it awkward to zoom in/out. I clicked on the sequon link or the position on the linear bars to zoom in (that was nice) but had to click on the original input query button to reset and zoom out. None of this is critical once you figure it out but perhaps this could be streamlined.

Are the conclusions about the tool and its performance adequately supported by the findings presented in the article?

Yes

Is the rationale for developing the new software tool clearly explained?

Yes

Is the description of the software tool technically sound?

Yes

Are sufficient details of the code, methods and analysis (if applicable) provided to allow replication of the software development and its use by others?

Yes

Is sufficient information provided to allow interpretation of the expected output datasets and any results generated using the tool?

Yes

Reviewer Expertise:

Molecular and Cellular Parasitology, Cell Biology, Glycobiology

I confirm that I have read this submission and believe that I have an appropriate level of expertise to confirm that it is of an acceptable scientific standard.

# Response

**Published:** 2022-02-09

**Authors:** Michele Tinti

**Affiliations:** Dundee University, UK

1) Thanks for spotting this inconsistency. We will fix it in the next version of the paper.

 2) I like this suggestion. I will add a polarity score to the feature panel; I'm thinking of using a sliding window of 5 amino acids.

 3) I used a slighter older screenshot than intended. I will replace the figure in the next version of the paper.

 4) Unfortunately, I don't think I can do any better for this functionality. I can zoom out with a two-finger click on my Mac laptop if it helps.
